# Thematic description of factors linked with extended-spectrum beta-lactamase-producing Enterobacteriaceae in humans

**DOI:** 10.14745/ccdr.v50i06a04

**Published:** 2024-06-28

**Authors:** Jamie Goltz, Carl Uhland, Sydney Pearce, Colleen Murphy, Carolee Carson, Jane Parmley

**Affiliations:** 1 Department of Population Medicine, Ontario Veterinary College, University of Guelph, Guelph, ON; 2 Centre for Food-borne, Infectious Diseases and Vaccination Programs Branch, Public Health Agency of Canada, Guelph, ON

**Keywords:** ESBL, Enterobacteriaceae, risk factors, humans, knowledge synthesis

## Abstract

**Background:**

Extended-spectrum beta-lactamase (ESBL)-producing Enterobacteriaceae are associated with serious antimicrobial-resistant infections in Canadians. Humans are exposed to ESBL-producing Enterobacteriaceae through many interconnected pathways. To better protect Canadians, it is important to generate an understanding of which sources and activities contribute most to ESBL exposure and infection pathways in Canada.

**Objective:**

The aims of this scoping review were to thematically describe factors potentially associated with ESBL-producing Enterobacteriaceae colonization, carriage and/or infection in humans from countries with a very high human development index and describe the study characteristics.

**Methods:**

Four databases (PubMed, CAB Direct, Web of Science, EBSCOhost) were searched to retrieve potentially relevant studies. Articles were screened for inclusion, and factors were identified, grouped thematically and described.

**Results:**

The review identified 381 relevant articles. Factors were grouped into 13 themes: antimicrobial use, animals, comorbidities and symptoms, community, demographics, diet and substance use, health care, household, occupation, prior ESBL colonization/carriage/infection, residential care, travel, and other. The most common themes reported were demographics, health care, antibiotic use and comorbidities and symptoms. Most articles reported factors in hospital settings (86%) and evaluated factors for ESBL-producing Enterobacteriaceae infections (52%).

**Conclusion:**

This scoping review provided valuable information about which factor themes have been well described (e.g., health care) and which have been explored less frequently (e.g., diet or animal contact). Themes identified spanned human, animal and environmental contexts and settings, supporting the need for a diversity of perspectives and a multisectoral approach to mitigating exposure to antimicrobial resistance.

## Introduction

Antimicrobial resistance (AMR) is a real and growing public health threat (1). Infections caused by extended-spectrum beta-lactamase (ESBL)-producing bacteria are a major concern because beta-lactam antibiotics are commonly used to treat a variety of infections, and some classes, such as third-generation cephalosporins and monobactams are listed as critically important for use in human medicine by the World Health Organization (2,3). Further, infections with ESBL-producing bacteria are associated with increased likelihood of severe illness and mortality and can result in treatment failures, which can lead to increased hospital-stay duration and hospital costs (4,5).

In 2018, it was reported that approximately one in four bacterial infections in Canada were resistant to first-line antibiotics, which led directly to approximately 14,000 deaths (5). Additionally, AMR has been reported to lead to negative socio-economic outcomes, including increased healthcare costs, loss of productivity, increased inequality and decreased trust in the government and public health agencies (5,6). Therefore, AMR consequences are far-reaching and have widespread implications to humans, animals and society.

The primary producers of ESBLs are Enterobacteriaceae, notably *Escherichia coli* and *Klebsiella pneumoniae*, and these bacteria are being increasingly identified in Canada, and worldwide (7–9). Extended-spectrum beta-lactamase-producing Enterobacteriaceae are widely dispersed among populations (9–11), including carriage or colonization within healthy individuals, and those with serious infections (e.g., urinary tract, bloodstream, pneumonia) (5,12,13).

Extended-spectrum beta-lactamase-producing Enterobacteriaceae have also been detected in companion animals, livestock, wildlife, water, soil, vegetables, meat and seafood, all of which can be possible sources of exposure for humans (11,14,15). Because of the variety of exposure pathways, a One Health approach that considers the interconnections between humans, animals, and their shared environments is required to cover the full scope of this growing public health threat (16,17).

Past systematic reviews have explored factors associated with ESBL-producing Enterobacteriaceae colonization and infections (13,18–27); however, systematic reviews are intentionally narrow in scope, providing knowledge on specific research questions. This project aimed to describe the breadth of factors previously reported to be associated with ESBL-producing Enterobacteriaceae in Canada or similar countries. This information could be used to inform various parallel projects within the Public Health Agency of Canada, such as the Integrated Assessment Model of Antimicrobial Resistance (iAM.AMR) project (15,28), and to help better understand Canadians’ exposure to antimicrobial-resistant bacteria. Therefore, the objectives of this scoping review were 1) to thematically describe factors potentially associated with ESBL-producing Enterobacteriaceae colonization, carriage and/or infection in humans from countries with a very high human development index, and 2) to describe the study characteristics.

## Methods

Below the methods are described in brief. For a full description of the methodology, refer to Goltz *et al.* (29).

### Protocol registration

An *a priori* protocol of this scoping review is available online. This review followed the methodological framework described by Arksey and O’Malley (30), and the guidelines of the Preferred Reporting Items for Systematic Reviews and Meta-Analyses (PRISMA) extension for Scoping Reviews (31).

### Search strategy

Search terms and databases searched are described in the protocol document. Four databases (PubMed, CAB Direct, Web of Science and EBSCOhost) were searched through the University of Guelph McLaughlin Library to retrieve potentially relevant articles. The search string for this review was adapted from Murphy *et al.* (32), with consultation from co-authors, in addition to a University of Guelph librarian. All databases were filtered to only include articles published in English. The initial search was completed in August 2020 and updated in August 2021.

Search results were uploaded into the EndNote X9.3.3 (Clarivate Analytics, Philadelphia, United States), deduplicated, and then uploaded to DistillerSR (Evidence Partners, Ottawa, Canada), for additional deduplication, eligibility screening and data extraction.

### Eligibility criteria

To meet the inclusion criteria, articles needed to be primary research, be from countries similar to Canada with a very high human development index (33), be written in English, and contain quantifying associations between factors and ESBL-producing Enterobacteriaceae colonization, carriage and/or infection in humans. No articles were excluded based on publication year, study population characteristics (e.g., age, sex or health status) or study setting (e.g., household or hospital). These inclusion criteria were selected because of the Canadian focus of this article, and therefore aimed to identify articles with Canadian and similar populations. Further, only English articles were included due to available language resources. Relevant systematic reviews and meta-analyses were excluded but their reference lists were used to identify additional articles that were not captured by the search.

### Selection of articles

The DistillerAI tool feature was used to screen titles/abstracts. The DistillerAI tool was trained by two reviewers using 226 articles. Once trained, all titles/abstracts were screened by the DistillerAI tool and a human reviewer. Title/abstract screening conflicts were resolved by a third human reviewer. Articles included based on title/abstract had the full text screened by two reviewers and conflicts were resolved through discussion by the two reviewers.

### Data charting

Following full text review, relevant data were charted using DistillerSR by a single reviewer. Data extracted included: publication year, study design, country region (based on World Health Organization regions) (34), data collection method (primary, e.g., questionnaire or interview; secondary, e.g., database or medical charts), sample setting (e.g., hospital), outbreak episode, age of participants, microorganisms evaluated, type of colonization, carriage, or infection evaluated and factor themes (n=13). A factor was defined as a measured observation (e.g., penicillin use) that was investigated for its relationship with ESBL-producing Enterobacteriaceae (32,35). Individual factors were grouped into 13 themes created through an iterative process informed by previous work (15). Themes were 1) antimicrobial use (i.e., antibacterial, antiviral, antifungal), 2) animals (i.e., contact with animals), 3) comorbidities and symptoms (i.e., conditions or presenting symptoms), 4) community (i.e., factors that occur in the community), 5) demographics, 6) food and consumption, 7) health care (i.e., factors that occur in a hospital setting or are related to receiving health care), 8) household (i.e., factors that occur at the home), 9) occupation (i.e., factors related to employment), 10) prior ESBL colonization/carriage/infection, 11) residential care (i.e., factors that occur in a residential setting such as a nursing home), 12) travel (i.e., factors related to international travel) and 13) other factors (i.e., factors that did not fall into a previously defined theme). If a factor belonged to more than one theme (e.g., patient took antibiotics while on vacation), it was recorded in all relevant themes (e.g., antimicrobial use and travel).

## Results

### Study screening and inclusion

**Figure 1:**
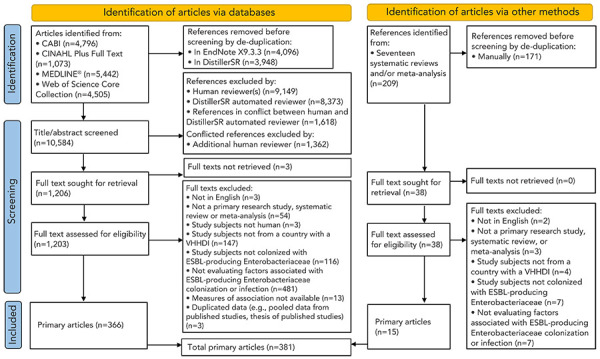
Flow diagram of the interface search, screening process and included articles to identify articles reporting risk factors for extended-spectrum beta-lactamase-producing Enterobacteriaceae in humans^a^ Abbreviations: ESBL, extended-spectrum beta-lactamase; VHHDI, very high human development index ^a^ Adapted from the Preferred Reporting Items for Systematic Reviews and Meta-Analyses (PRISMA) flow diagram factor themes ((36))

After deduplication, 10,584 eligible records were identified. Following screening (abstract/title, full text), 366 articles were included. Screening also identified 17 systematic reviews and/or meta-analyses and 15 additional articles were identified through review of their reference lists. Therefore, 381 articles were included in this review, published between 1991 and August 5, 2021 (**Figure 1**).

Across the 381 included articles, factors were grouped into 13 themes: health care (n=325 articles), antimicrobial use (n=325), demographics (n=319), comorbidities and symptoms (n=307), residential care (n=76), travel (n=76), prior ESBL colonization/carriage/infection (n=44), food and consumption (n=44), household (n=29), occupation (n=29), animal (n=25), community (n=11) and other (n=146) (additional details available upon request). Each theme covered a wide range of risk factors associated with ESBL-producing Enterobacteriaceae (**Table 1**).

**Table 1:** Description of factors represented by the factor themes for colonization, carriage and/or infection with extended-spectrum beta-lactamase-producing Enterobacteriaceae reported by the articles included in this review

Factor theme	Factor categories	Factor examples
Antimicrobial use	Antibiotic, antiparasitic, antiviral, antifungal use	Penicillin use Amoxicillin-clavulanate use Fluconazole use
	Status of antimicrobials	Mother given antibiotics before delivery Admitted on antibiotics Inadequate empirical antibiotic treatment
Animal	Animal contact	Cat owner Living with dogs Farm animal contact
	Animal lifestyle	Pet given antibiotics ESBL in pigs Companion animal eats raw meat
Community	Community activities	Public swimming/bathing in freshwater or seawater Playing on a sports team Daycare attendance
Comorbidities and symptoms	History of a medical condition	AIDS Cancer Diabetes
	Comorbidity scores	Charlson comorbidity index ICU chronic disease score Sequential organ failure assessment score
	Symptoms	Blood pressure Fever Septic shock
Demographics	Demographic information	Age Ethnicity Language spoken
Health care	Healthcare setting	Admitted from home Admission to emergency department Prior ICU
	Healthcare setting risks	ESBL-positive prior room occupant Hospital length of stay Hand disinfectant in the patient’s room
	Procedures or treatments	Chemotherapy Surgery Acid suppressor use
Household	Household members	Family member is a carrier (mother, father, sister) Children younger than 12 years old in the household Household member took antibiotics
	Household setting risks	Shared use of towels Distance to nearest broiler farm
Residential care	Residential care stay	Nursing home residence Long-term care facility stay
	Residential care setting risks	Use of shared bathroom Staff training in hand hygiene Existence of a preferential list of antibiotics
Food and consumption	Food and water type	Chicken consumption Seafood consumption Bottled water
	Food and water source	Purchased from market/shop Own produce/local farmer Central water supply
	Food and water handling	Sterilized feeding bottles Regular/sometimes hand washing before food preparation Dishcloth use longer than one day
	Substance consumption	Alcohol Smoking Illicit drugs
Prior ESBL colonization/carriage/infection	Prior ESBL-producing organism	Prior ESBL colonization Prior ESBL infection
Occupation	Occupation type	Veterinarian Farmer Caregiver
	Occupation setting risks	Average number of hours working on the pig farm per week Contact with patient’s excretions Assistance in patient’s wound care
Travel	Travel risks	Visited other country Health care abroad Accommodation type (e.g., camping, house, hotel, with locals)
Other	Acquisition/onset location	Community acquisition Acquired prior to admission Nosocomial onset
	Time of acquisition	Season Year of sample
	Details about bacteria	Resistant genes Polymicrobial information

Abbreviations: ESBL, extended-spectrum beta-lactamase; ICU, intensive care unit

### Study characteristics

A summary description of the articles is reported in **Table 2**. Of the 381 articles included, 378 were observational study designs, and three were experimental. Most of the studies (n=235) were conducted in European Region countries, including six multinational studies (Table 2). Seven studies were conducted in Canada.

**Table 2:** Study characteristics of the included articles

Study characteristics	Number of articles	
	n	%
**Study design**		
Observational	378	99
Experimental	3	1
**Country region**		
European Region^a^	235	62
Western Pacific Region	78	20
Region of the Americas	60	16
Eastern Mediterranean Region	8	2
**Age group**		
Adults/young adults	125	33
Children	40	11
Neonates/newborns/infants	19	5
Multiple defined age groups (e.g., children and adults)	15	4
Elderly	14	4
Undefined	168	44
**Setting where samples were obtained^b^**		
Hospital	328	86
Non-hospital health care	27	7
Community	22	6
Residential care facilities	16	4
**Outbreak**		
No	363	95
Yes	18	5
**Factor data collection method**		
Secondary data (e.g., databases, medical charts)	201	53
Primary data (e.g., questionnaire, interview)	76	20
Multiple data collection methods (i.e., primary and secondary data)	27	7
Unclear	77	20
**Microorganisms evaluated**		
Enterobacteriaceae^c^	154	40
*Escherichia coli*	78	20
*Klebsiella pneumoniae*	42	11
*Enterobacter cloacae*	4	1
*Klebsiella* spp.	2	1
*Proteus mirabilis*	2	1
*Providencia stuartii*	1	1
Other^d^	98	26

^a^ Six multinational articles within the European Region

^b^ Eleven articles sampled from multiple of these different sample settings

^c^ Studies evaluating Enterobacteriaceae, Enterobacterales or Enterobacteria

^d^ Different combinations of bacteria species not described broadly as Enterobacteriaceae (e.g., *K*. *pneumoniae* and *E. coli*, bacterial isolates/cultures, gram-negative bacteria)

Over half (56%) of all articles reported data for specific age groups with the most common being adults/young adults (33%). Eighteen articles (5%) reported factors as part of an ESBL-producing Enterobacteriaceae outbreak (all in hospital settings). For most studies (53%), data were reported from secondary data sources (e.g., databases, medical charts), with 20% from primary data sources (e.g., questionnaires, interviews) and 7% from both primary and secondary data sources. For 20% of the studies, it was unclear how the data were obtained (Table 2).

Articles often reported factors for Enterobacteriaceae (40%), but many reported specific microorganisms including *E. coli* (20%), *K. pneumoniae* (11%), *Enterobacter cloacae* (1%), *Klebsiella* spp. (1%), *Proteus mirabilis* (1%), and *Providencia stuartii* (1%). Other articles sought to report different combinations of Enterobacteriaceae species (e.g., *Klebsiella* spp. and *E. coli*) (Table 2).

**Figure 2:**
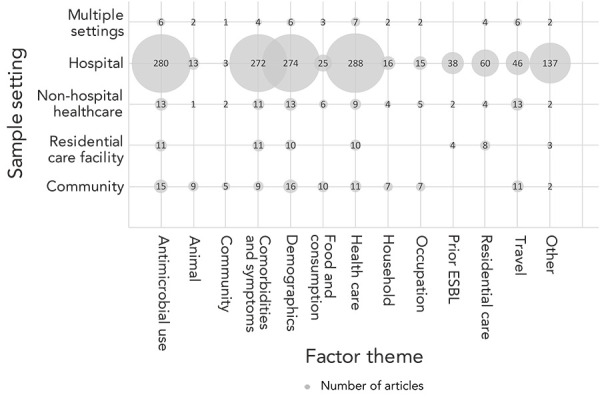
Factor themes reported in the articles by study sample setting for colonization/carriage and/or infection with extended-spectrum beta-lactamase-producing Enterobacteriaceae^a^ Abbreviation: ESBL, extended-spectrum beta-lactamase ^a^ The number of corresponding articles correlates to the bubble size

Most articles were performed in hospital settings (86%), followed by non-hospital healthcare settings (7%), community settings (6%), and residential care facilities (4%). Eleven of these articles were sampled from multiple of these different sample settings (Table 2). Overall, the highest number of articles identified for each factor theme were those that had performed their study in hospital settings, except for the community theme (**Figure 2**).

**Figure 3:**
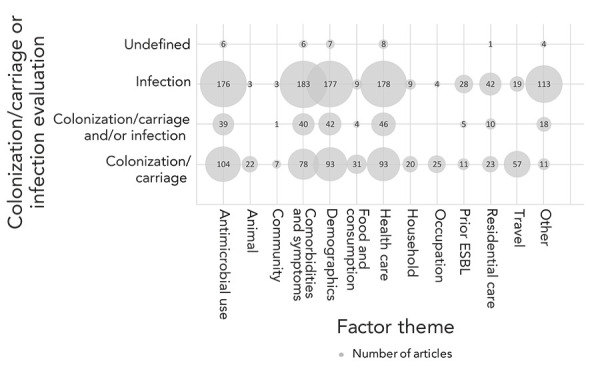
Factor themes reported in the articles by the study evaluation of colonization/carriage or infection for colonization/carriage and/or infection with extended-spectrum beta-lactamase-producing Enterobacteriaceae^a^ Abbreviation: ESBL, extended-spectrum beta-lactamase ^a^ The number of corresponding articles correlates to the bubble size

Articles reported factors for 1) infection (52%), 2) colonization/carriage (33%), and 3) colonization/carriage/infection (13%) (**Table 3**). Factors potentially associated with ESBL-producing Enterobacteriaceae infections were reported in over half of the articles (52%) (mostly bloodstream infections or urinary tract infections). More articles identified factors for infection than colonization/carriage (**Figure 3**), especially for the factor themes antimicrobial use, demographics, comorbidities/symptoms and health care. Colonization/carriage was reported in a third of the articles (33%), with most focused on gastrointestinal carriage. Animal, community, food and consumption, household, occupation and travel themes were more frequently reported for colonization/carriage (Figure 3). For eight articles (2%) it was unclear whether the study was reporting colonization/carriage or infection.

**Table 3:** Description of reported extended-spectrum beta-lactamase-producing Enterobacteriaceae outcomes reported in the included articles

Colonization/carriage and/or infection details	Number of articles^a^	
	n	%
**Infection**	**198**	**52**
Bacteremia/bloodstream infection	75	20
Urinary tract infection	52	14
Non-specific cultures (e.g., general surveillance or database records)	49	13
Acute pyelonephritis	5	1
Acute bacterial prostatitis	2	1
Bacteremia/bloodstream infection and urinary tract infection	1	1
Bacteremia/bloodstream infection, urinary tract infection and catheter-associated infection	1	1
Bacteremic spontaneous bacterial peritonitis	1	1
Catheter-associated urinary tract infection	1	1
Complicated cystitis	1	1
Foot infection	1	1
Genital tract infections	1	1
Peritonitis	1	1
Pneumonia	1	1
Sepsis	1	1
Spontaneous bacterial peritonitis	1	1
Sternal wound infection	1	1
Urinary tract infection/acute pyelonephritis	1	1
Urosepsis	1	1
Ventilator-associated pneumonia	1	1
**Colonization/carriage**	**125**	**33**
Gastrointestinal (e.g., fecal, stool, rectal, peri-rectal)	110	29
Non-specific cultures (e.g., general surveillance or database records)	5	1
Gastrointestinal and nasal	2	1
Gastrointestinal and vaginal	2	1
Gastrointestinal, vaginal and nasopharyngeal	1	1
Gastrointestinal, nasal and navel	1	1
Gastrointestinal, nasal, oropharyngeal and urine	1	1
Gastrointestinal, nasal and throat	1	1
Skin	1	1
Urinary	1	1
**Colonization/carriage and/or infection**	**51**	**13**
Non-specific colonization/carriage and/or non-specific infection	38	10
Urinary colonization/carriage and/or urinary tract infection	6	1
Gastrointestinal colonization/carriage and/or non-specific infection	5	1
Respiratory colonization/carriage and/or infection	1	1
Urinary colonization/carriage and/or urinary tract infection, cystitis and pyelonephritis	1	1
**Unclear**	**8**	**2**
Non-specific isolation	7	2
Urinary isolation	1	1

^a^ Four articles had two extended-spectrum beta-lactamase-producing Enterobacteriaceae outcome evaluations

Many comparison groups were reported (**Table 4**). The most common was an ESBL-positive Enterobacteriaceae culture compared with an ESBL-negative Enterobacteriaceae culture (n=171). Twenty articles reported two comparator groups (e.g., case-case-control studies).

**Table 4:** Reporting of outcome comparisons among articles for colonization/carriage and/or infection with extended-spectrum beta-lactamase-producing Enterobacteriaceae in the included articles

Positive outcome (e.g., cases)	Negative outcome (e.g., controls)	Number of articles^a^	
		n	%
ESBL-positive for Enterobacteriaceae culture (e.g., ESBL-producing *E. coli* urine culture)	ESBL-negative for the same explicitly defined Enterobacteriaceae culture (e.g., non-ESBL-producing *E. coli* urine culture)^b^	171	45
ESBL-producing Enterobacteriaceae colonization/carriage positive (e.g., ESBL-producing *E. coli* fecal sample)	Negative for same explicitly defined ESBL-producing Enterobacteriaceae colonization/carriage (e.g., negative for ESBL-producing *E. coli* fecal sample)^c^	119	31
ESBL-producing Enterobacteriaceae infection positive (e.g., ESBL-producing *E. coli* UTI)	Negative for same explicitly defined ESBL-producing Enterobacteriaceae infection (e.g., negative for ESBL-producing *E. coli* UTI)^d^	34	9
ESBL-producing Enterobacteriaceae colonization/carriage and/or infection (e.g., ESBL-producing Enterobacteriaceae colonization/carriage or infection)	Negative for same explicitly defined ESBL-producing Enterobacteriaceae colonization/carriage and/or infection (e.g., negative for ESBL-producing Enterobacteriaceae colonization/carriage or infection)^e^	32	8
ESBL positive for Enterobacteriaceae culture (e.g., ESBL-producing Enterobacteriaceae urine culture)	ESBL negative for a combination of explicitly defined Enterobacteriaceae and non-Enterobacteriaceae of the same culture (e.g., non-ESBL-producing Enterobacteriaceae and non-ESBL-producing non-Enterobacteriaceae urine culture)^f^	16	4
ESBL positive for Enterobacteriaceae culture (e.g., ESBL-producing *E. coli* urine culture)	ESBL negative for bacteria that was not explicitly defined of the same culture (e.g., non-ESBL-producing bacterial urine culture)^g^	7	2
Developed an ESBL-producing Enterobacteriaceae infection	Positive for ESBL-producing Enterobacteriaceae colonization/carriage	6	2
CTX-M-producing Enterobacteriaceae	Different genotype producing Enterobacteriaceae (e.g., TEM or SHV-producing Enterobacteriaceae)	5	1
Positive for ESBL-producing Enterobacteriaceae culture (e.g., ESBL-producing *E. coli* blood culture)	ESBL-negative with a different explicitly defined Enterobacteriaceae or non-Enterobacteriaceae bacteria culture (e.g., non-ESBL-producing *K. pneumoniae* or *Pseudomonas* spp. blood culture)	5	1
Positive for ESBL-producing Enterobacteriaceae culture acquired in a specified setting (e.g., community-acquired *E. coli* UTI)	Positive for ESBL-producing Enterobacteriaceae culture acquired in a different specified setting (e.g., hospital-acquired *E. coli* UTI)	2	1
Positive for ESBL-producing Enterobacteriaceae colonization/carriage in combination with an ESBL-producing Enterobacteriaceae infection	Positive for ESBL-producing Enterobacteriaceae colonization/carriage in combination with an infection not caused by ESBL-producing Enterobacteriaceae	2	1
ESBL-positive for Enterobacteriaceae culture (e.g., ESBL-producing *E. coli* urine)	ESBL-positive for Enterobacteriaceae from a different culture (e.g., ESBL-producing *E. coli* blood)	1	1
ESBL-positive Enterobacteriaceae culture (e.g., ESBL-producing *E. coli* blood culture)	The same culture with any other bacteria than the compared ESBL-positive Enterobacteriaceae strain (i.e., cultures could be negative or positive for any bacteria blood culture except ESBL-producing *E. coli*)	1	1

Abbreviations: *E. coli*, *Escherichia coli*; ESBL, extended-spectrum beta-lactamase; UTI, urinary tract infection

^a^ Twenty articles provided two comparisons

^b^ Control groups were non-ESBL producers but may have other resistance-susceptibility profiles

^c^ Control groups were negative for the same ESBL-producing Enterobacteriaceae colonization/carriage; however, the controls were positive or negative for the presence of other Enterobacteriaceae or non-Enterobacteriaceae cultures

^d^ Control groups were negative for the same ESBL-producing Enterobacteriaceae infection; however, the controls were positive or negative for the presence of other Enterobacteriaceae or non-Enterobacteriaceae cultures

^e^ Control groups were negative for the same ESBL-producing Enterobacteriaceae colonization/carriage and/or infection. However, the presence of other Enterobacteriaceae or non-Enterobacteriaceae cultures were not explicitly reported

^f^ Control group explicitly reported Enterobacteriaceae in combination with non-Enterobacteriaceae families (e.g., *E. coli*, *Klebsiella* spp., and *Pseudomonas* spp.)

^g^ Control group did not explicitly report bacterial species within the study; therefore, it was unclear whether the control group included only Enterobacteriaceae cultures or whether non-Enterobacteriaceae species cultures were included

## Discussion

In this scoping review, we identified 381 articles reporting factors for ESBL-producing Enterobacteriaceae. Most of the included articles were published in the last 10 years, likely corresponding to the urgency to understand the growing rates of human acquisition of ESBL-producing Enterobacteriaceae and the exponential growth of scientific publications generally (8,37–39). It is noteworthy that most articles focused on factors related to antimicrobial use, comorbidities/symptoms, demographics and health care, and that only a small proportion of identified articles reported factors associated with animal contact, community, and food and consumption; mainly related to colonization/carriage of ESBL-producing Enterobacteriaceae. Although there were fewer articles that reported these themes, they may provide important information as previous articles have suggested that animal contact, food consumption and household or community transmission may play a role in ESBL-producing Enterobacteriaceae exposure (11,17,40–42). It is unclear whether the individual factors that were most frequently reported in these articles were in fact more often associated with ESBL-producing Enterobacteriaceae (i.e., had larger measures of association), whether they had been evaluated and reported more frequently than others, or whether studies evaluating these factors were better funded.

Study setting may be an explanation for the larger number of articles on antimicrobial use, comorbidities/symptoms, demographics and health care factors reported. Most articles were conducted in hospital settings and over half of the articles used secondary sources of information (e.g., medical records or databases). This setting and source combination may have been selected on account of the relative ease of accessibility to the data. Factors associated with resistant infections in hospitals are major concerns, and therefore are an important area of research. Although some factors reported from hospital settings may be connected to those in the community settings (e.g., taking medication), factors reported from hospital settings may not be representative of factors from community settings (e.g., populations, comorbidities, varying activities). Thus, the results from studies conducted in hospital settings are not generalizable to other settings.

This review identified studies where the subjects were sampled from countries with a very high human development index (33) as we were interested in factors relevant to the Canadian context. Most studies were conducted in the European Region (n=235), followed by the Western Pacific (n=78), the Americas (n=60) and the Eastern Mediterranean (n=8). Only seven studies were performed in Canada; however, a large body of literature was collected that can be used to understand the existing knowledge of factors associated with acquiring ESBL-producing Enterobacteriaceae in similar populations. Although these countries have similarities, differences in policies and practices may limit the generalizability of the data specifically to Canada.

Several articles reported similar factors for Enterobacteriaceae infection, regardless of their AMR status, including comorbidities, demographics and health care (43–45). Factors associated with ESBL-producing Enterobacteriaceae colonization or infection may be related to bacterial traits rather than distinguishing between susceptible and resistant bacteria, which is important because interventions that target the pathogen, regardless of the resistance, are likely effective at reducing both resistant and susceptible strains. This highlights the importance of selecting the appropriate comparator (control group) for the intended research question and interpretation of findings. Many different outcome comparators were identified in our review. Each comparison combination provides different information that contributes to a better overall understanding of factors associated with ESBL-producing Enterobacteriaceae.

This article reports the breadth of factors associated with ESBL-producing Enterobacteriaceae reported in the literature. Many references frequently reported demographic factors (e.g., age and ethnicity) and groups that may be particularly vulnerable. While these factors cannot be modified, they can be used to identify particularly vulnerable groups for which interventions can be targeted. Other articles reported modifiable factors (e.g., food, travel, antimicrobial use), which can be targeted as interventions and potentially implemented immediately (e.g., food-related interventions), noting dependencies on feasibility and cost, whereas others may require more gradual, multi-pronged solutions (e.g., reducing comorbidities). A multidisciplinary approach to address feasible health-promoting strategies and the complex nature of AMR with multiple drivers is necessary.

Work is currently underway to better describe the factors identified in these articles. This will provide the number of factors reported per study and quantitative data reported for these factors (i.e., the strength and direction of association between the factor and ESBL-producing Enterobacteriaceae). Further, factors from this review will be used to populate models within the iAM.AMR project (15,28) to improve our understanding of the pathways of human exposure to ESBL-producing Enterobacteriaceae. This information will help to inform which human characteristics, behaviours and actions impact the probability of becoming colonized or infected with ESBL-producing Enterobacteriaceae and to identify which factors to prioritize for interventions. This information will be valuable for understanding how to advise Canadians about mitigating their probability of acquiring resistant bacteria and reducing the negative health impacts associated with infection.

## Limitations

Articles were identified from select online databases, omitting research from grey literature. This may have introduced a publishing bias, as findings that were not disseminated through peer-reviewed publications were not reviewed for inclusion (e.g., theses and dissertations, government reports) and articles with null, negative or inconclusive findings are less likely to be published (46). Language bias was a consideration as the review was constrained to English-language articles; however, the impact of this bias was likely negligible as approximately 98% of science publications are written in English (47,48).

Another limitation included single reviewer data extraction on account of resource limitations. Multiple individuals extracting study data reduces errors and misclassification bias (49). To mitigate these types of errors and to identify errors in data extraction, the authors were involved in both data collection development and analysis.

Lastly, the grouping of factors into themes evolved during data extraction. Grouping factors into themes was challenging because of differences in terminology used, the populations studied, and definitions applied. Combining data from different studies was onerous due to heterogeneity of the study data (e.g., same variable measured on different scales, missing data) (50). Terms, including carriage and colonization, were not standardized across studies and were used interchangeably; therefore, some data had to be combined (e.g., colonization and/or carriage) or captured as “unclear.”

## Conclusion

This review synthesized evidence from a large collection of articles reporting factors associated with ESBL-producing Enterobacteriaceae colonization, carriage and/or infections in humans within very high human development index countries. Factors were reported in many different settings, age groups and organisms, and using different outcome comparison groups. This variability between studies highlighted the need for transparent or, where possible, harmonized reporting of methods to allow for appropriate interpretations and comparisons between the factors reported. Overall, studies conducted in hospital settings predominated and the most common factor themes reported were antimicrobial use, comorbidities/symptoms, demographics and health care. Articles reporting animal contact, food consumption/practices and activities in the community were not as numerous and thus limited information about these factors were identified. There is a need for more studies examining factors associated with ESBL-producing Enterobacteriaceae in the community, which have been identified as being of concern (6,8).

This scoping review synthesized knowledge about potential sources and activities that affect the risk of human exposure to ESBL-producing Enterobacteriaceae. Factor themes identified spanned human, animal and environmental contexts and settings support the need for a diversity of perspectives and a multisectoral approach to AMR. The results of this article will help guide recommendations to reduce the risk of acquiring ESBL-producing Enterobacteriaceae for Canadians, as well as other similar countries, while considering numerous sources of exposure in various settings. These results will also guide future research for activities and in settings that are understudied.

## References

[1] Public Health Agency of Canada. Pan-Canadian Action Plan on Antimicrobial Resistance. Ottawa, ON: PHAC; 2024. https://www.canada.ca/en/public-health/services/publications/drugs-health-products/pan-canadian-action-plan-antimicrobial-resistance.html

[2] Public Health Agency of Canada. Canadian Antimicrobial Resistance Surveillance System Report - Update 2020. Ottawa, ON: PHAC; 2020. https://www.canada.ca/en/public-health/services/publications/drugs-health-products/canadian-antimicrobial-resistance-surveillance-system-2020-report.html

[3] World Health Organization. Critically Important Antimicrobials for Human Medicine: 6th revision. Geneva, CH: WHO; 2019. https://www.who.int/publications/i/item/9789241515528

[4] MacKinnon MC, Sargeant JM, Pearl DL, Reid-Smith RJ, Carson CA, Parmley EJ, McEwen SA. Evaluation of the health and healthcare system burden due to antimicrobial-resistant Escherichia coli infections in humans: a systematic review and meta-analysis. Antimicrob Resist Infect Control 2020;9(1):200. 10.1186/s13756-020-00863-x 33303015 PMC7726913

[5] Council of Canadian Academies. When Antibiotics Fail. Ottawa, ON: CCA; 2019. https://cca-reports.ca/reports/the-potential-socio-economic-impacts-of-antimicrobial-resistance-in-canada/

[6] Prestinaci F, Pezzotti P, Pantosti A. Antimicrobial resistance: a global multifaceted phenomenon. Pathog Glob Health 2015;109(7):309–18. 10.1179/2047773215Y.0000000030 26343252 PMC4768623

[7] Lagacé-Wiens PR, Adam HJ, Poutanen S, Baxter MR, Denisuik AJ, Golden AR, Nichol KA, Walkty A, Karlowsky JA, Mulvey MR, Golding G, Hoban DJ, Zhanel GG; Canadian Antimicrobial Resistance Alliance (CARA) and CANWARD. Trends in antimicrobial resistance over 10 years among key bacterial pathogens from Canadian hospitals: results of the CANWARD study 2007-16. J Antimicrob Chemother 2019;74 Suppl 4:iv22–31. 10.1093/jac/dkz284 31505648

[8] Bezabih YM, Sabiiti W, Alamneh E, Bezabih A, Peterson GM, Bezabhe WM, Roujeinikova A. The global prevalence and trend of human intestinal carriage of ESBL-producing Escherichia coli in the community. J Antimicrob Chemother 2021;76(1):22–9. 10.1093/jac/dkaa399 33305801

[9] Doi Y, Iovleva A, Bonomo RA. The ecology of extended-spectrum β-lactamases (ESBLs) in the developed world. J Travel Med 2017;24 suppl_1:S44–51. 10.1093/jtm/taw102 28521000 PMC5731446

[10] Pitout JD, Nordmann P, Laupland KB, Poirel L. Emergence of Enterobacteriaceae producing extended-spectrum beta-lactamases (ESBLs) in the community. J Antimicrob Chemother 2005;56(1):52–9. 10.1093/jac/dki166 15917288

[11] Mughini-Gras L, Dorado-García A, van Duijkeren E, van den Bunt G, Dierikx CM, Bonten MJ, Bootsma MC, Schmitt H, Hald T, Evers EG, de Koeijer A, van Pelt W, Franz E, Mevius DJ, Heederik DJ; ESBL Attribution Consortium. Attributable sources of community-acquired carriage of Escherichia coli containing β-lactam antibiotic resistance genes: a population-based modelling study. Lancet Planet Health 2019;3(8):e357–69. 10.1016/S2542-5196(19)30130-5 31439317

[12] Pitout JD. Infections with extended-spectrum β-lactamase-producing enterobacteriaceae: changing epidemiology and drug treatment choices. Drugs 2010;70(3):313–33. 10.2165/11533040-000000000-00000 20166768

[13] Karanika S, Karantanos T, Arvanitis M, Grigoras C, Mylonakis E. Fecal Colonization With Extended-spectrum Beta-lactamase-Producing Enterobacteriaceae and Risk Factors Among Healthy Individuals: A Systematic Review and Metaanalysis. Clin Infect Dis 2016;63(3):310–8. 10.1093/cid/ciw283 27143671

[14] Huijbers PM, Blaak H, de Jong MC, Graat EA, Vandenbroucke-Grauls CM, de Roda Husman AM. Role of the Environment in the Transmission of Antimicrobial Resistance to Humans: A Review. Environ Sci Technol 2015;49(20):11993–2004. 10.1021/acs.est.5b02566 26355462

[15] Primeau C. Exploring the contributions of genotypic, phenotypic, social and qualitative data sources to our understanding of antimicrobial resistance in Canada. University of Guelph, 2020. http://hdl.handle.net/10214/17935

[16] McEwen SA, Collignon PJ. Antimicrobial Resistance: a One Health Perspective. Microbiol Spectr 2018;6(2): 10.1128/microbiolspec.ARBA-0009-2017 29600770 PMC11633550

[17] Schmithausen R, Schulze-Geisthoevel SV, Heinemann C, Bierbaum G, Exner M, Petersen B, Steinhoff-Wagner J. Reservoirs and Transmission Pathways of Resistant Indicator Bacteria in the Biotope Pig Stable and along the Food Chain: A Review from a One Health Perspective. Sustainability 2018;10(11):3967. 10.3390/su10113967

[18] Alevizakos M, Karanika S, Detsis M, Mylonakis E. Colonisation with extended-spectrum β-lactamase-producing Enterobacteriaceae and risk for infection among patients with solid or haematological malignancy: a systematic review and meta-analysis. Int J Antimicrob Agents 2016;48(6):647–54. 10.1016/j.ijantimicag.2016.08.021 27746102

[19] Larramendy S, Deglaire V, Dusollier P, Fournier JP, Caillon J, Beaudeau F, Moret L. Risk Factors of Extended-Spectrum Beta-Lactamases-Producing *Escherichia coli* Community Acquired Urinary Tract Infections: A Systematic Review. Infect Drug Resist 2020;13:3945–55. 10.2147/IDR.S269033 33177845 PMC7650195

[20] Butcher CR, Rubin J, Mussio K, Riley LW. Risk Factors Associated with Community-Acquired Urinary Tract Infections Caused by Extended-Spectrum β-Lactamase-Producing Escherichia coli: a Systematic Review. Curr Epidemiol Rep 2019;6(5):300–9. 10.1007/s40471-019-00206-4

[21] Detsis M, Karanika S, Mylonakis E. ICU Acquisition Rate, Risk Factors, and Clinical Significance of Digestive Tract Colonization With Extended-Spectrum Beta-Lactamase-Producing Enterobacteriaceae: A Systematic Review and Meta-Analysis. Crit Care Med 2017;45(4):705–14. 10.1097/CCM.0000000000002253 28157141

[22] Flokas ME, Detsis M, Alevizakos M, Mylonakis E. Prevalence of ESBL-producing Enterobacteriaceae in paediatric urinary tract infections: A systematic review and meta-analysis. J Infect 2016;73(6):547–57. 10.1016/j.jinf.2016.07.014 27475789

[23] Furuya-Kanamori L, Stone J, Yakob L, Kirk M, Collignon P, Mills DJ, Lau CL. Risk factors for acquisition of multidrug-resistant Enterobacterales among international travellers: a synthesis of cumulative evidence. J Travel Med 2020;27(1):taz083. 10.1093/jtm/taz083 31691808

[24] Hendrik TC, Voor In ’t Holt AF, Vos MC. Clinical and Molecular Epidemiology of Extended-Spectrum Beta-Lactamase-Producing Klebsiella spp.: A Systematic Review and Meta-Analyses. PLoS One 2015;10(10):e0140754. 10.1371/journal.pone.0140754 26485570 PMC4617432

[25] Hu YJ, Ogyu A, Cowling BJ, Fukuda K, Pang HH. Available evidence of antibiotic resistance from extended-spectrum β-lactamase-producing Enterobacteriaceae in paediatric patients in 20 countries: a systematic review and meta-analysis. Bull World Health Organ 2019;97(7):486–501B. 10.2471/BLT.18.225698 31258218 PMC6593334

[26] Li X, Xu X, Yang X, Luo M, Liu P, Su K, Qing Y, Chen S, Qiu J, Li Y. Risk factors for infection and/or colonisation with extended-spectrum β-lactamase-producing bacteria in the neonatal intensive care unit: a meta-analysis. Int J Antimicrob Agents 2017;50(5):622–8. 10.1016/j.ijantimicag.2017.06.027 28733213

[27] Wuerz TC, Kassim SS, Atkins KE. Acquisition of extended-spectrum beta-lactamase-producing Enterobacteriaceae (ESBL-PE) carriage after exposure to systemic antimicrobials during travel: systematic review and meta-analysis. Travel Med Infect Dis 2020;37:101823. 10.1016/j.tmaid.2020.101823 32755674

[28] Chapman B, Phillips C. The IAM.AMR Project Documentation. iAM.AMR; 2019. https://docs.iam.amr.pub/en/latest/

[29] Goltz J. Investigating Factors Associated with Extended-Spectrum Beta-Lactamase-Producing Enterobacteriaceae Colonization and/or Infection in Humans: A One Health Approach. University of Guelph; 2022. https://atrium.lib.uoguelph.ca/server/api/core/bitstreams/1e4611d9-eb58-439d-902f-f424f29ad8e9/content

[30] Arksey H, O’Malley L. Scoping studies: towards a methodological framework. Int J Soc Res Methodol Theory Pract. 2005;8(1):19–32. 10.1080/1364557032000119616

[31] Tricco AC, Lillie E, Zarin W, O’Brien KK, Colquhoun H, Levac D, Moher D, Peters MD, Horsley T, Weeks L, Hempel S, Akl EA, Chang C, McGowan J, Stewart L, Hartling L, Aldcroft A, Wilson MG, Garritty C, Lewin S, Godfrey CM, Macdonald MT, Langlois EV, Soares-Weiser K, Moriarty J, Clifford T, Tunçalp Ö, Straus SE. PRISMA Extension for Scoping Reviews (PRISMA-ScR): checklist and Explanation. Ann Intern Med 2018;169(7):467–73. 10.7326/M18-0850 30178033

[32] Murphy CP, Carson C, Smith BA, Chapman B, Marrotte J, McCann M, Primeau C, Sharma P, Parmley EJ. Factors potentially linked with the occurrence of antimicrobial resistance in selected bacteria from cattle, chickens and pigs: A scoping review of publications for use in modelling of antimicrobial resistance (IAM.AMR Project). Zoonoses Public Health 2018;65(8):957–71. 10.1111/zph.12515 30187682

[33] United Nations Development Programme. Human Development Report 2019. Beyond income, beyond averages, beyond today: Inequalities in human development in the 21st century. New York, NY: UNDP; 2019. https://hdr.undp.org/content/human-development-report-2019

[34] World Health Organization. Countries, Geneva, CH: WHO; 2024. https://www.who.int/countries

[35] Phillips C, Chapman B, Agunos A, Carson CA, Parmley EJ, Reid-Smith RJ, Smith BA, Murphy CP. A scoping review of factors potentially linked with antimicrobial-resistant bacteria from turkeys (iAM.AMR Project). Epidemiol Infect 2022;150:e153. 10.1017/S0950268822001224 35843720 PMC9428905

[36] Page MJ, McKenzie JE, Bossuyt PM, Boutron I, Hoffmann TC, Mulrow CD, Shamseer L, Tetzlaff JM, Akl EA, Brennan SE, Chou R, Glanville J, Grimshaw JM, Hróbjartsson A, Lalu MM, Li T, Loder EW, Mayo-Wilson E, McDonald S, McGuinness LA, Stewart LA, Thomas J, Tricco AC, Welch VA, Whiting P, Moher D. The PRISMA 2020 statement: an updated guideline for reporting systematic reviews. BMJ 2021;372(71):n71. 10.1136/bmj.n71 33782057 PMC8005924

[37] Denisuik AJ, Karlowsky JA, Adam HJ, Baxter MR, Lagacé-Wiens PR, Mulvey MR, Hoban DJ, Zhanel GG; Canadian Antimicrobial Resistance Alliance (CARA) and CANWARD. Dramatic rise in the proportion of ESBL-producing Escherichia coli and Klebsiella pneumoniae among clinical isolates identified in Canadian hospital laboratories from 2007 to 2016. J Antimicrob Chemother 2019;74 Suppl 4:iv64–71. 10.1093/jac/dkz289 31505647

[38] Woerther PL, Burdet C, Chachaty E, Andremont A. Trends in human fecal carriage of extended-spectrum β-lactamases in the community: toward the globalization of CTX-M. Clin Microbiol Rev 2013;26(4):744–58. 10.1128/CMR.00023-13 24092853 PMC3811232

[39] Bornmann L, Haunschild R, Mutz R. Growth rates of modern science: a latent piecewise growth curve approach to model publication numbers from established and new literature databases. Humanit Soc Sci Commun 2021;8(224): 10.1057/s41599-021-00903-w

[40] Riccio ME, Verschuuren T, Conzelmann N, Martak D, Meunier A, Salamanca E, Delgado M, Guther J, Peter S, Paganini J, Martischang R, Sauser J, de Kraker ME, Cherkaoui A, Fluit AC, Cooper BS, Hocquet D, Kluytmans JA, Tacconelli E, Rodriguez-Baño J, Harbarth S; MODERN WP2 study group. Household acquisition and transmission of extended-spectrum β-lactamase (ESBL) -producing Enterobacteriaceae after hospital discharge of ESBL-positive index patients. Clin Microbiol Infect 2021;27(9):1322–9. 10.1016/j.cmi.2020.12.024 33421572

[41] Haverkate MR, Platteel TN, Fluit AC, Cohen Stuart JW, Leverstein-van Hall MA, Thijsen SF, Scharringa J, Kloosterman RC, Bonten MJ, Bootsma MC. Quantifying within-household transmission of extended-spectrum β-lactamase-producing bacteria. Clin Microbiol Infect 2017;23(1):46.e1–7. 10.1016/j.cmi.2016.08.021 27596534

[42] Toombs-Ruane LJ, Benschop J, French NP, Biggs PJ, Midwinter AC, Marshall JC, Chan M, Drinković D, Fayaz A, Baker MG, Douwes J, Roberts MG, Burgess SA. Carriage of Extended-Spectrum-Beta-Lactamase- and AmpC Beta-Lactamase-Producing Escherichia coli Strains from Humans and Pets in the Same Households. Appl Environ Microbiol 2020;86(24):e01613–20. 10.1128/AEM.01613-20 33036993 PMC7688229

[43] Meatherall BL, Gregson D, Ross T, Pitout JD, Laupland KB. Incidence, risk factors, and outcomes of Klebsiella pneumoniae bacteremia. Am J Med 2009;122(9):866–73. 10.1016/j.amjmed.2009.03.034 19699383

[44] Laupland KB, Gregson DB, Church DL, Ross T, Pitout JD. Incidence, risk factors and outcomes of Escherichia coli bloodstream infections in a large Canadian region. Clin Microbiol Infect 2008;14(11):1041–7. 10.1111/j.1469-0691.2008.02089.x 19040476

[45] Villafuerte D, Aliberti S, Soni NJ, Faverio P, Marcos PJ, Wunderink RG, Rodriguez A, Sibila O, Sanz F, Martin-Loeches I, Menzella F, Reyes LF, Jankovic M, Spielmanns M, Restrepo MI; GLIMP Investigators. Prevalence and risk factors for Enterobacteriaceae in patients hospitalized with community-acquired pneumonia. Respirology 2020;25(5):543–51. 10.1111/resp.13663 31385399

[46] Paez A. Gray literature: an important resource in systematic reviews. J Evid Based Med 2017;10(3):233–40. 10.1111/jebm.12266 28857505

[47] Moher D, Pham B, Lawson ML, Klassen TP. The inclusion of reports of randomised trials published in languages other than English in systematic reviews. Health Technol Assess 2003;7(41):1–90. 10.3310/hta7410 14670218

[48] Gordin MD. Scientific Babel. How Science Was Done Before and After Global English. Chicago: University of Chicago Press; 2015.

[49] Higgins JP, Thomas J, Chandler J, Cumpston M, Li T, Page MJ, Welch VA, editors. Cochrane Handbook for Systematic Reviews of Interventions version 6.4. Cochrane, 2023. www.training.chochrane.org/handbook

[50] Rao SR, Graubard BI, Schmid CH, Morton SC, Louis TA, Zaslavsky AM, Finkelstein DM. Meta-analysis of survey data: application to health services research. Health Serv Outcomes Res Methodol 2008;8:98–114. 10.1007/s10742-008-0032-0

